# National HIV and HCV Screening Rates for Hospitalized People who Use Drugs Are Suboptimal and Heterogeneous Across 11 US Hospitals

**DOI:** 10.1093/ofid/ofae204

**Published:** 2024-04-16

**Authors:** Leo Knudsen Westgard, Taisuke Sato, William S Bradford, Ellen F Eaton, Finlay Pilcher, Andrew J Hale, Devika Singh, Marlene Martin, Ayesha A Appa, Jaimie P Meyer, Melissa B Weimer, Lydia A Barakat, Uriel R Felsen, Matthew J Akiyama, Jessica P Ridgway, Emily D Grussing, Kinna Thakarar, Amy White, John Mutelayi, Martin Krsak, Brian T Montague, Ank Nijhawan, Harini Balakrishnan, Laura R Marks, Alysse G Wurcel

**Affiliations:** Tufts Medical Center, Boston, Massachusetts, USA; Tufts Medical Center, Boston, Massachusetts, USA; University of Alabama at Birmingham, Birmingham, Alabama, USA; University of Alabama at Birmingham, Birmingham, Alabama, USA; Larner College of Medicine, University of Vermont, Burlington, Vermont, USA; Larner College of Medicine, University of Vermont, Burlington, Vermont, USA; University of Vermont Medical Center, Burlington, Vermont, USA; Larner College of Medicine, University of Vermont, Burlington, Vermont, USA; University of Vermont Medical Center, Burlington, Vermont, USA; Zuckerberg San Francisco General Hospital, San Francisco, California, USA; University of California, San Francisco, California, USA; University of California, San Francisco, California, USA; Yale School of Medicine, New Haven, Connecticut, USA; Yale University School of Public Health, New Haven, Connecticut, USA; Yale School of Medicine, New Haven, Connecticut, USA; Yale New Haven Hospital, New Haven, Connecticut, USA; Yale School of Medicine, New Haven, Connecticut, USA; Montefiore Medical Center, Bronx, New York, USA; Albert Einstein College of Medicine, Bronx, New York, USA; Montefiore Medical Center, Bronx, New York, USA; Albert Einstein College of Medicine, Bronx, New York, USA; University of Chicago, Chicago, Illinois, USA; Tufts Medical Center, Boston, Massachusetts, USA; Tufts University School of Medicine, Boston, Massachusetts, USA; Tufts University School of Medicine, Boston, Massachusetts, USA; Maine Medical Center Research Institute, Portland, Maine, USA; Maine Medical Center, Portland, Maine, USA; Maine Medical Partners Adult Infectious Diseases, South Portland, Maine, USA; Maine Medical Center Research Institute, Portland, Maine, USA; Maine Medical Center Research Institute, Portland, Maine, USA; University of Colorado School of Medicine, Denver, Colorado, USA; University of Colorado School of Medicine, Denver, Colorado, USA; University of Texas Southwestern Medical Center, Dallas, Texas, USA; University of Texas Southwestern Medical Center, Dallas, Texas, USA; Washington University in St. Louis School of Medicine, St. Louis, Missouri, USA; Tufts Medical Center, Boston, Massachusetts, USA; Tufts University School of Medicine, Boston, Massachusetts, USA

**Keywords:** HIV testing, injection drug use, people who inject drugs, hospitalization, hepatitis C testing

## Abstract

**Background:**

To end the HIV and hepatitis C virus (HCV) epidemics, people who use drugs (PWUD) need more opportunities for testing. While inpatient hospitalizations are an essential opportunity to test people who use drugs (PWUD) for HIV and HCV, there is limited research on rates of inpatient testing for HIV and HCV among PWUD.

**Methods:**

Eleven hospital sites were included in the study. Each site created a cohort of inpatient encounters associated with injection drug use. From these cohorts, we collected data on HCV and HIV testing rates and HIV testing consent policies from 65 276 PWUD hospitalizations.

**Results:**

Hospitals had average screening rates of 40% for HIV and 32% for HCV, with widespread heterogeneity in screening rates across facilities. State consent laws and opt-out testing policies were not associated with statistically significant differences in HIV screening rates. On average, hospitals that reflexed HCV viral load testing on HCV antibody testing did not have statistically significant differences in HCV viral load testing rates. We found suboptimal testing rates during inpatient encounters for PWUD. As treatment (HIV) and cure (HCV) are necessary to end these epidemics, we need to prioritize understanding and overcoming barriers to testing.

To achieve the United States’ *Ending the HIV Epidemic* goal of reducing new HIV infections by 75% by 2025, widespread screening for HIV is essential [1]. Around 40% of new infections are transmitted by people who do not know they have HIV [2]. HIV screening limits transmission of the virus and is the first step to linking patients to life-saving care. Moreover, HIV testing is cost-effective at every health interaction, from ambulatory care to the emergency department to hospitalization [3–5].

People who use drugs (PWUD) are a key population to screen for HIV and hepatitis C virus (HCV), especially people who inject drugs (PWID). Depending on the city and type of survey, about 20% to 65% of PWID report sharing syringes, which is a risk factor for HIV and HCV transmission [6–10]. HIV screening rates for PWID are low compared with other at-risk populations, further contributing to the spread of HIV and HCV in injection drug use and sexual networks [11]. Only 55% of PWID reported receiving guideline-recommended annual HIV testing in 2018 [12], and fewer than half of PWID with HIV in the United States are virally suppressed [13]. Prospective cohort studies in the United States show broad ranges of HCV antibody prevalence from 30% to 70% among PWUD [14–16]. Inpatient screening for HCV is not explicitly addressed in the Centers for Disease Control and Prevention (CDC) guidelines [17]; however, given the high rates of HCV prevalence in people who use drugs and the availability of curative, well-tolerated, all-oral HCV treatment, HCV testing is recommended as a best practice for all people who inject drugs who are admitted to the hospital [18, 19].

Inpatient hospitalizations present an opportunity to reach PWUD, offer substance use disorder treatment, and provide testing and other preventive care for infectious diseases. PWUD are, on average, 7 times more likely to be admitted to a hospital than the general population [1], and PWUD utilize primary care less than half as frequently as people who do not use drugs [2]. Furthermore, hospitalizations for PWID specifically continue to rise from increased rates of serious injection-related infections like endocarditis and osteomyelitis [20, 21]. HIV and HCV testing should be offered to all people who are hospitalized. However, despite recommendations for inpatient HIV screening, a Boston hospital reported that only about 10% of hospitalized PWID between 2017 and 2020 were tested for HIV. People who identified as Black and Hispanic/Latinx had decreased odds of HIV testing [22, 23]. Another study of hospitalized PWID with serious infections seen by infectious disease providers found that 86% received testing for HCV and 88% received testing for HIV [24].

The goal of this study was to evaluate if US hospitals are sufficiently screening PWUD for HIV and HCV during inpatient hospitalizations. We hypothesized that HIV and HCV testing rates would be heterogeneous, and HIV testing rates would be discordant with CDC guidance suggesting testing during hospitalization. We also hypothesized that state-mandated requirements for verbal consent may be associated with lower HIV testing rates.

## METHODS

**Figure 1.**
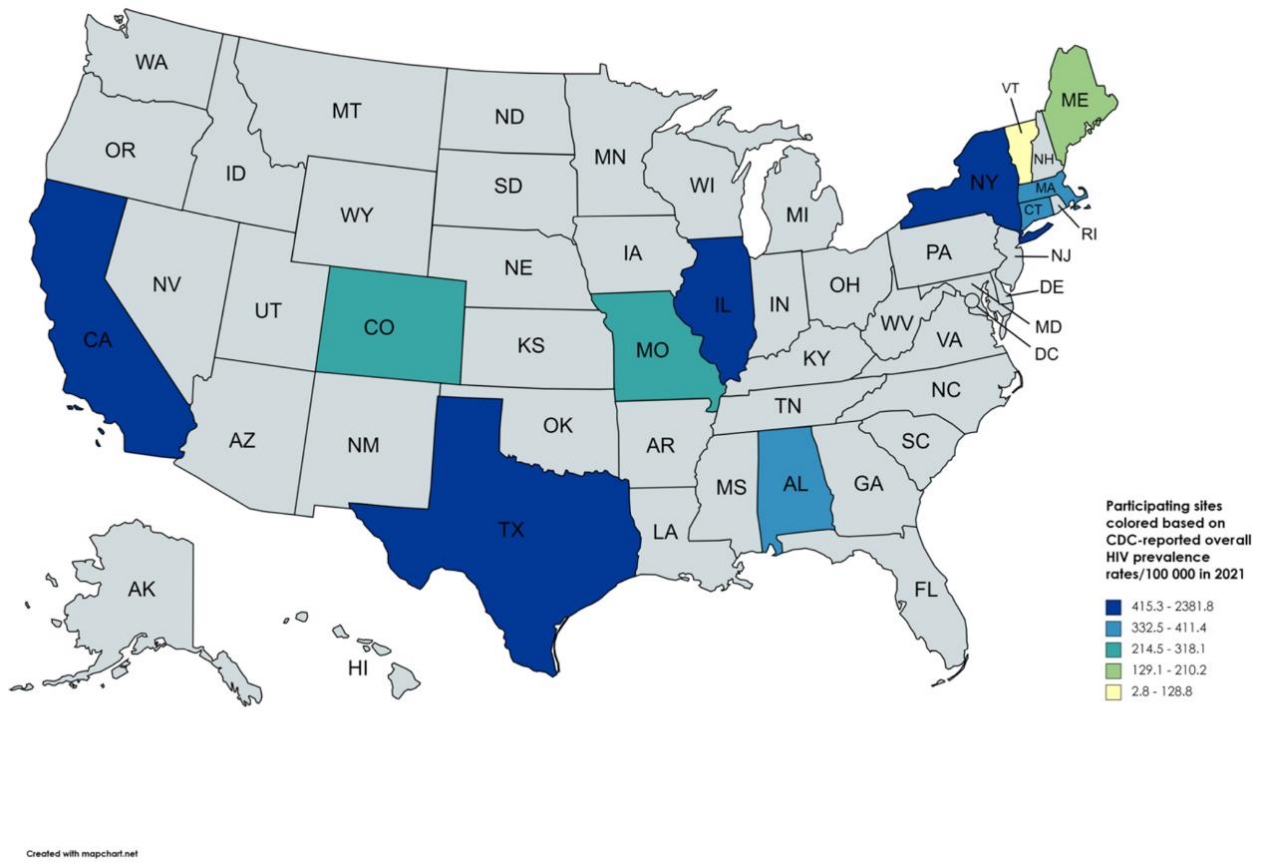
HIV prevalence in the 11 states included in the analysis (2021). Source: New HIV diagnoses and people with diagnosed HIV in the US and dependent areas by area of residence, 2021 [26].

This research group emerged from previous collaborations facilitated by the Infectious Disease Society of America working group on infections related to opioid use disorder. Eleven sites were selected where collaborators were available to extract HIV testing data, with geographic diversity in mind. After consulting with their local IRBs and gaining approval, researchers and clinicians collaborated on data collection (Figure 1) [25, 26]. The Tufts research team (L.K.W., T.S., E.G., A.G.W.) created a protocol for data collection. Collaborators from each site created a cohort of inpatient encounters from 1/1/2020 to 4/1/2022 with a diagnosis code potentially indicative of injection drug use (Table 1). We also included 2 sites (CT and TX) that were able to pull data on preexisting cohorts of patients who utilized their hospitals’ addiction medicine services. The unit of analysis was hospitalization, not the individual patient, because each hospitalization represents an opportunity to offer HIV and/or HCV testing.

**Table 1.** ICD-10-CM Parent Codes Indicative of IDU

ICD-10-CM Code	Description
F11.x	Opioid-related disorders
F14.x	Cocaine-related disorder
F15.x	Other stimulant-related disorder
F19.x	Other psychoactive substance use
T40.0x	Poisoning by adverse effect of and underdosing of opium
T40.1x	Poisoning by and adverse effect of heroin
T40.2x	Poisoning by adverse effect of and underdosing of other opioids
T40.3x	Poisoning by adverse effect of and underdosing of methadone
T40.4x	Poisoning by adverse effect of and underdosing of other synthetic narcotics
T40.5x	Poisoning by adverse effect of and underdosing of cocaine
T40.6x	Poisoning by adverse effect of and underdosing of other and unspecified narcotics
T43.6x	Poisoning by adverse effect of and underdosing of psychostimulants

Source: Authors’ analysis of codes based off clinical experience.

Abbreviations: ICD-10-CM, International Classification of Diseases, 10th Revision, Clinical Modification; IDU, injection drug use.

In addition to information about the hospitals themselves, each site was asked to provide the following information on their cohort of encounters: total number of hospitalizations, HIV antigen/antibody test completed, positive HIV test, HCV antibody test completed, positive HCV antibody test, and HCV viral load test completed (HCV viral load was not reported by all sites; Table 2). From these data, testing rates and test positivity rates were calculated using the total number of hospitalizations in the cohort as a denominator. Each site was asked for HIV screening and consent policies of the hospital in addition to if they reflex HCV viral load testing from HCV antibody testing. To ensure standardization and simplify data collection, we did not exclude hospitalizations of patients who had already been diagnosed with infection or had previously received a test for either HIV or HCV. To evaluate the impact of state-mandated consent requirements on testing rates, we conducted a difference-in-means Student *t* test comparing testing rates segmented by different hospitals’ testing policies. Finally, we measured the correlation between testing rates and test positivity.

**Table 2.** HIV and HCV Testing and Positivity Rates of PWUD Admitted to 11 Hospitals and Hospital Systems (January 2020 to April 2022)

Region Hospital/State	No. of PWUD Admissions	HIV Testing Completed, %	(+) HIV Test, %	HCV AB Completed, %	HCV VL Completed, %	(+) HCV Test, %	Verbal Consent Required	Opt-Out HIV Testing	Reflex HCV VL From AB Testing
Midwest									
Barnes Jewish Hospital, MO	5242	41	5.6	44	16	24	No	Yes	Yes
University of Chicago, IL	760	59	3.8	21	6.4	32	No^a^	Yes	Yes
Northeast									
Montefiore Medical Center, NY	10 667	17	4.3	17	9.0	32	No^b^	No	Yes
Tufts Medical Center, MA	2217	24	NA	22	7.5	61	Yes	No	No
UVM Medical Center, VT	2434	15	0.0	20	NA	52	Yes	Yes	Yes
Maine Medical Center, ME	1254	9.6	0.0	15^c^	NA^c^	42^c^	Yes	No	Yes^d^
Yale-New Haven Health, CT^e^	3428	35	5.5	37	NA	65	No	Yes	Yes
West									
San Fran. General Hospital, CA	3918	78	3.6	49	11	17	Yes	Yes	No
UCHealth, CO	27 985	72	1.9	55	NA	28	No	No	No
South									
UAB Medicine, AL	6913	53	NA	50	22	NA	Yes^a^	Yes	Yes
Parkland Hospital, TX^e^	458	38	1.7	18	11	62	No^a^	No	No
	Mean	40	2.9	32	10	41			
	(SD)	23	2.1	15	6.7	19			

Source: Authors’ analysis of data from host institutions.

Abbreviations: AB, antibody; HCV, hepatitis C virus; PWUD, people who use drugs; VL, viral load.

^a^HIV test consent is in initial treatment plans; patients are notified and can refuse.

^b^Facilities require consent, even if not oral/written. NY requires notification of HIV testing with the opportunity to decline.

^c^HCV AB test rates apply hospital-wide, except Maine Medical Center: antibody or viral load.

^d^Able to be ordered but not always ordered.

^e^Data sets from hospitalized addiction consultation patients.

## RESULTS

There were 65 276 hospitalizations of PWUD from 11 states included in this study. Hospitals had an average HIV screening rate of 40% and an average HCV Ab screening rate of 32% for encounters fitting inclusion criteria (Table 2). For both viruses, there was widespread heterogeneity in testing rates across facilities, with standard deviations of 23% and 15%, respectively. Five of the 11 hospitals required verbal consent for HIV testing. HIV testing rates were on average lower for hospitals that required consent and hospitals that did not have opt-out testing policies. However, those differences were not statistically significant (Table 3). Likewise, HCV viral load testing rates did not significantly differ between hospitals that reflex HCV viral load testing from HCV antibody testing and those that do not. The average test positivity across hospitals was 2.9% for HIV tests and 41% for HCV antibody tests. There was a negative correlation between test positivity and antibody testing rates for both HIV (−0.177) and HCV (−0.340).

**Table 3.** HIV and HCV Testing Rates Segmented by Hospitals’ Testing Policies (January 2020–April 2022)

	Mean HIV Testing Rate, %	*P* Value
Verbal consent required		.607
Yes	36	
No	44	
Opt-out testing policy		.319
Yes	47	
No	32	

	Mean HCV AB Testing Rate, %	*P* Value
Reflex HCV VL from AB testing		.508
Yes	29	
No	36	

	Mean HCV VL Testing Rate, %	*P* Value
Reflex HCV VL from AB testing		.45
Yes	13	
No	10	

Source: Authors’ analysis of data from host institutions.

Abbreviations: AB, antibody; HCV, hepatitis C virus; VL, viral load.

## DISCUSSION

In this multicenter study of hospitalizations of PWUD across 11 states, HIV and HCV testing rates were heterogeneous and discordant with best practices for comprehensive care. Testing rates for HCV were lower than those for HIV, with widespread heterogeneity across hospitals, regardless of consent requirements. The HCV screening rates are particularly alarming given that there is an established cure for HCV infection and that separate consent is not required for testing. To reap the full benefits of screening for both infections, testing must be paired with strategies that ensure adequate linkage to and retention in care such as patient navigation services [27–29].

While we did not find statistically significant associations between consent requirements or opt-out testing policies and HIV testing rates, small nonsignificant differences in testing rates indicate that consent requirements and opt-in testing policies may limit testing rates. More research with larger sample sizes is needed to confirm this hypothesis. Likewise, insignificant differences in HCV viral load testing rates between hospitals that reflex viral load testing from antibody testing and those that do not may be due to our small sample size. It is also possible that sites included in our study without reflex policies sufficiently use viral load tests to confirm HCV diagnoses. While this correlation was not particularly strong for either infection, testing rates were on average lower for hospitals with higher test positivity rates.

Currently, 20 states require documented verbal consent for HIV screening, exceeding regular requirements to receive care [30]. Consent requirements have been previously shown to be a barrier to HIV testing [31–33], and clinicians have reported feeling unprepared and too busy to consent patients for HIV [34, 35]. Inability to consent patients in altered mental states during admission often precludes HIV testing as well [36]. In states where stringent guidelines on HIV consent persist, hospitals should streamline the consent process, clearly designate whose job it is to ask for consent, educate providers on how to do so, and integrate consent into the general admission consent for treatment, when possible [37].

Rates of utilization of medication for opioid use disorder (MOUD) and pre-exposure prophylaxis to prevent HIV (PrEP) in PWUD, similar to HIV and HCV testing, are suboptimal [38, 39]. Protocolized care, such as a “PWID service bundle” as proposed by the CDC [40], should include testing for HIV, hepatitis B and C, and sexually transmitted infections (STIs); treatment for infectious diseases; vaccination for hepatitis A and B; syringe service resources; medications for opioid use disorder; naloxone distribution; and PrEP. Bundled testing (eg, HIV + STIs, HIV + HCV, bundled reflex testing for HCV) has been shown to increase testing in nontraditional infectious diseases health care settings [41, 42], including in clinics that serve unhoused populations [43, 44]. Clinical decision support systems, such as electronic health record (EHR) reminders for patients with a history of SUD who have not recently received a test and including consent for HIV in the nursing intake process, could also be used to support bundled care [45].

Barriers to HIV testing include stigmatization and lack of focus on preventative care in hospitalized settings [32, 33]. For many clinicians, HIV and HCV testing are considered outpatient tests as the majority of US HIV tests are conducted in outpatient clinics [46]. However, PWUD are less likely to visit health care clinicians in these settings [47]. Thus, there may not be opportunities for outpatient testing for HIV and HCV for this population [48, 49]. Most people who are hospitalized are admitted for acute medical problems unrelated to HIV or HCV [50]. Whereas hospitals have an economic incentive to rapidly address acute issues, preventative services including testing for HIV and HCV are less lucrative and deprioritized [51]. Indeed, many PWUD are uninsured, which means that HIV testing may be uncompensated. Thus, inpatient providers may underprioritize HIV and HCV testing.

HCV testing rates were lower than HIV testing rates. This may be because clinicians and patients view HCV as a chronic disease that is common in PWUD and not an urgent issue, because PWUD feel they are at low risk, or because providers incorrectly believe HCV treatment should only be offered after a period of abstinence from drug use [52]. Thus, patients admitted with complications of active substance use may not fall within clinicians’ routine practice for HCV testing and treatment. Another reason for lower HCV testing rates may be because the CDC began to recommend routine HCV screening in 2020, 14 years after it was recommended for HIV [53].

There are several limitations to this analysis. Only a small number of urban academic medical centers with physician researchers already studying HIV and HCV in PWUD were included in this study. These sites may not be representative of nor generalizable to all hospitals in the United States. Testing rates are likely to be lower in rural settings with fewer resources and restricted access to care [54]. Testing rates for hospitalized people were likely impacted by the significant disruptions in clinical protocols coinciding with the COVID-19 pandemic. For example, testing may have decreased due to competing demands or challenges obtaining consent among hospitalized patients during periods with widespread isolation protocols and minimized noncritical face-to-face patient contact. Notably, our data extend past the time of the initial pandemic bursts of hospitalizations. Also, it is worth studying testing during periods of COVID-19 because harm reduction services were also disrupted [55], leading to increased rates of overdose and hospitalizations for PWUD [56]. This may have heterogeneously affected participating sites. Alternatively, HIV testing rates increased in 1 hospital for PWUD during the first wave of COVID-19 [22]. Not all PWUD are PWID, so we also may have overestimated the number of people at risk for infections transmitted by needles. Similarly, some encounters may have been for patients who had already screened positive for HIV or HCV or recently received an HIV or HCV test, meaning another test may not have been warranted. Additionally, high rates of positivity at some sites may reflect increased rates of repeat or confirmatory HIV testing among patients already diagnosed with HIV during the COVID-19 pandemic to facilitate linkage and engagement during a period that saw significant disruptions in care. Given that HCV is much more common than HIV, more people may have already been diagnosed, meaning no retesting was offered. Another limitation is that HCV confirmatory testing is often not reflexive and requires a subsequent laboratory encounter, which would not have been captured in our analysis. For some patients, HCV viral load testing may have been done in the absence of an HCV antibody test if the patient reported a history of HCV or prior treatment.

## CONCLUSIONS

This study adds to the literature by reporting site-level screening rates across a geographically diverse cohort of numerous US hospitals and showing relatively low HIV and HCV screening in hospitalized PWUD. Testing PWUD during inpatient admissions is a missed opportunity to link this marginalized population to care and prevent comorbidities and new infections. Robust, collaborative interventions and funding are imperative to mitigating future waves of HIV and HCV outbreaks in PWID. We have the tools; it is time to wield them.
